# Foliar magnesium application improves sweet corn yield: boosting nutrient uptake and grain carbohydrate under dense planting condition

**DOI:** 10.3389/fpls.2025.1499391

**Published:** 2025-05-09

**Authors:** Delian Ye, Zexun Yu, Jiajie Chen, Sifan Wei, Zifeng Zhang, Siyu Huang, Da Su, Tiedong Liu, Muhammad Atif Muneer

**Affiliations:** ^1^ Key Laboratory of Ministry of Education for Genetics, Breeding and Multiple Utilization of Crops, College of Agriculture, Fujian Agriculture and Forestry University, Fuzhou, China; ^2^ Key Laboratory of Biological Breeding for Fujian and Taiwan Crops, Ministry of Agriculture and Rural Affairs, Fujian Agriculture and Forestry University, Fuzhou, China; ^3^ International Magnesium Institute, College of Resources and Environmental Sciences, Fujian Agriculture and Forestry University, Fuzhou, China

**Keywords:** sweet corn, foliar spraying magnesium, magnesium uptake, magnesium remobilization, grain filling, fresh ear yield

## Abstract

Magnesium (Mg) plays a critical role in regulating yield and grain quality in corn. However, its management is often overlooked in cultivation, particularly under high-density planting conditions, where intensified nutrient competition can exacerbate Mg deficiency. To address the knowledge gap in Mg management strategies, we conducted three-season field trials (2021-2022) evaluating foliar spraying with different concentrations of Mg fertilizer under dense planting conditions. The results showed that foliar spraying with 4% Mg significantly increased sweet corn fresh ear yield, ear weight, grain fresh weight, and grains per ear, while reducing the abortion rate compared to the foliar water spraying (CK) treatment across all three seasons. Foliar spraying with 4% Mg markedly increased dry matter, N, K, Ca, and Mg accumulation in sweet corn over two seasons in 2022. The ear leaf Soil Plant Analysis Development (SPAD) value under 4% Mg treatment increased by an average of 6.9% and 9.3% at the R1 and R3 stages, respectively, compared to CK treatment. Although 21.1% of total Mg accumulation (16.9 kg ha^−^¹) was acquired at post-silking, vegetative remobilization contributed minimally to total Mg (< 5%). The 4% Mg treatment significantly increased the grain filling rate at 7 and 12 days after silking, and increased grain sucrose, fructose, and glucose concentrations by an average of 14.4%, 2.7%, and 9.3%, respectively, at 27 days after silking compared to CK treatment. Based on these findings, we propose a practical Mg fertilization guideline for high-density sweet corn cultivation: three foliar applications of 4% MgSO_4_·7H_2_O, totaling 8.78 kg Mg ha^-1^. This strategy improves nutrient uptake, enhances grain carbohydrate accumulation, and supports yield optimization without compromising grain quality.

## 1 Introduction

Sweet corn (*Zea mays* var. *saccharata*) is widely recognized for its dual role as a nutritious staple food source and a popular vegetable (Singh et al., 2014). In recent years, its cultivation has expanded across China, primarily driven by growing consumer demand and its economic profitability for farmers (Xue et al., 2023). Given China’s limited arable land resources, improving sweet corn yield per unit area is essential. While increased planting density represents a crucial strategy for boosting corn yield, this practice often negatively impacts grains per ear and grain weight (Dhaliwal and Williams, 2019; Ye et al., 2023a, 2023b). This phenomenon can be attributed to two interdependent mechanisms. First, high-density planting accelerates leaf senescence, diminishing photosynthetic capacity and disrupting photosynthate allocation patterns (Lan et al., 2024). Second, restricted root elongation and reduced root-to-shoot ratios impair nutrient acquisition efficiency, intensifying intra-plant nutrient competition under dense planting (Shao et al., 2024). Consequently, developing optimal fertilization strategies to enhance grains per ear and grain weight under high planting density sweet corn cultivation remains a critical research priority.

Magnesium (Mg) is an essential nutrient that affects yield and quality formation but is often overlooked in crop cultivation (Cakmak and Kirkby, 2008). Mg enhances crop growth by improving photosynthesis (via chlorophyll stabilization), carbohydrate partitioning, and nitrogen (N) use efficiency through modulation of nitrate transporters (Farhat et al., 2016; Peng et al., 2020). It also increases resilience to abiotic stress by regulating reactive oxygen species homeostasis and activating antioxidant enzymes (Tian et al., 2021). Mg deficiency is prevalent in tropical and subtropical acidic soils, where high rainfall accelerates leaching, and crop harvesting removes soil Mg reserves (Ishfaq et al., 2022). Additionally, Chinese farmers commonly apply excessive amounts of N and potassium (K) fertilizers while neglecting Mg fertilizers. This unbalanced fertilization practice, coupled with soil Mg deficiency, exacerbates the antagonistic interaction between Mg²^+^ and other competing cations, further limiting Mg availability (Fageria, 2001; Gransee and Führs, 2013; Wang et al., 2020). Southeast China, a major sweet corn cultivation region, faces significant issues of soil Mg deficiency and imbalanced fertilization management (Ishfaq et al., 2022). As a result, Mg deficiency is a critical limiting factor for increasing sweet corn yields in Southeast China.

Mg fertilizer application serves as a critical agronomic practice for mitigating Mg deficiency and increasing yield (Wang et al., 2020). For instance, soil-applied Mg fertilizer significantly increases field corn yield and Mg accumulation in northeast China (Zhang et al., 2020). Meanwhile, soil application of Mg fertilizer has little effect on increasing yield under non-adverse conditions (Grzebisz, 2013). In contrast, foliar Mg fertilization offers distinct advantages over soil application. This method enables rapid nutrient uptake by evading soil constraints such as cation antagonism and restricted root proliferation, directly delivering Mg through foliar tissues (Grzebisz, 2013; Xie et al., 2021; Niu et al., 2021). Moreover, foliar spraying ensures timely delivery of Mg during critical growth stages, boosting photosynthesis, enhancing field corn resistance to stress, and ultimately improving yield (Rodrigues et al., 2021). Nevertheless, previous studies indicate proper foliar Mg fertilizer rates vary with crop types and agroecological regions (Kibria et al., 2021). Consequently, establishing quantifiable relationships between foliar Mg dosage and sweet corn yield remains imperative. However, research on sweet corn in acidic soils under field conditions in China remains limited.

Nutrient uptake fundamentally governs corn yield, with optimized fertilization strategies enhancing both nutrient uptake and yield potential (Ye et al., 2023a; Bindraban et al., 2015). While elucidating Mg uptake is critical for rational Mg fertilization, substantial genotypic variation in Mg absorption persists among field corn genotypes despite documented yield and nutrient accumulation improvements through Mg supplementation (Ciampitti and Vyn, 2013; El-Dissoky et al., 2017; Zhang et al., 2020). This knowledge gap necessitates quantitative characterization of Mg uptake patterns in sweet corn to inform precision Mg management. In addition, grain weight is one of the most crucial yield components, and grain carbohydrates play important roles in grain weight formation (Paponov et al., 2005). Recent evidence indicates that dense planting maintains grain carbohydrate concentration but reduces grain weight in sweet corn (Ye et al., 2023b). Although Mg application improves grain weight in wheat and field corn (Zhang et al., 2021a; Ceylan et al., 2016), its effects on grain weight and carbohydrates in sweet corn still require further research.

Mg deficiency is common for sweet corn cultivation in Southeast China, and dense planting deteriorates sweet corn yield and quality traits. However, little information focuses on whether foliar Mg spraying can alleviate the impact of high density on sweet corn. We assumed that Mg application can increase the yield of sweet corn under high planting by improving nutrient absorption and sugar synthesis. Therefore, this study aims to investigate the effect of foliar spraying Mg on fresh ear yield, Mg accumulation and allocation, grain filling, and carbohydrate concentration of sweet corn under field conditions in Southeast China, providing a basis for the scientific application of Mg fertilizer.

## 2 Materials and methods

### 2.1 Experimental location

**Figure 1:**
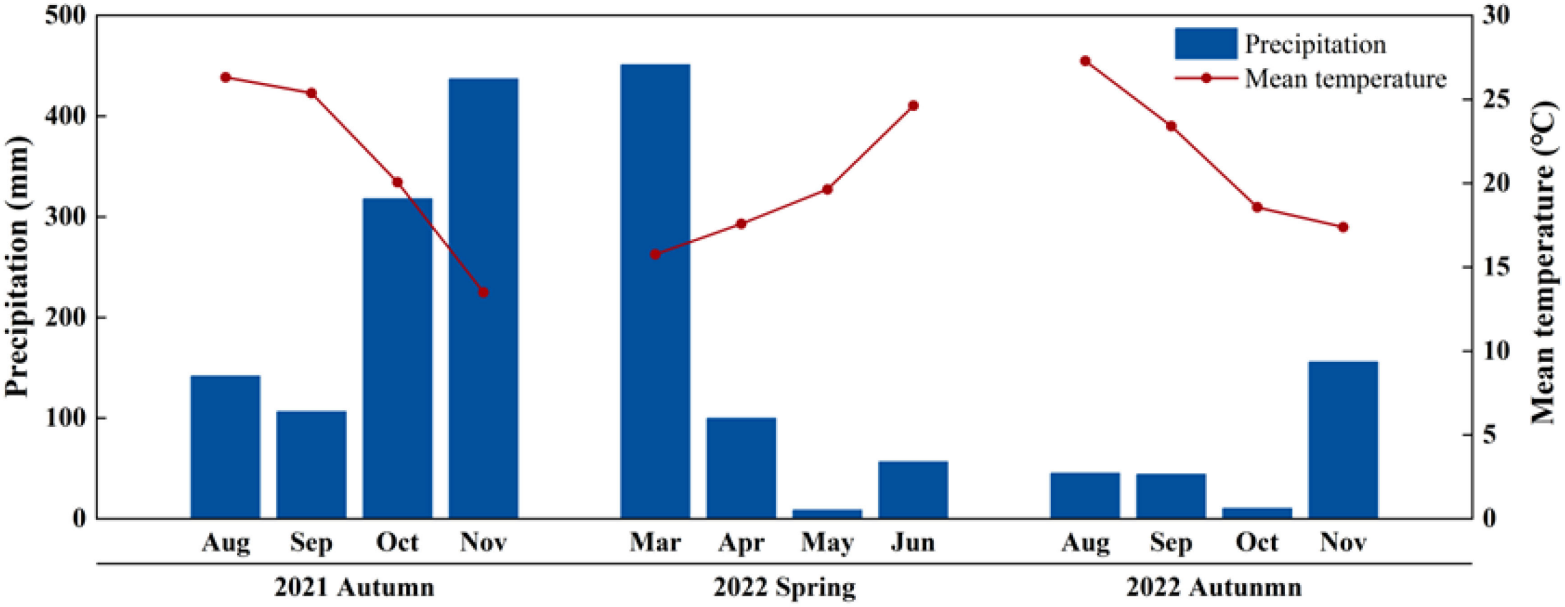
The monthly precipitation and mean temperature during the 2021–2022 growing season.

The field experiment was conducted in 2021 (autumn season) and 2022 (spring and autumn season) at Yuanfeng Farm (26°8′ N, 118°44′ E) in Minqing County, Fujian Province, Southeast China. This region has a subtropical humid monsoon climate; the mean temperature and total precipitation during successive growing seasons were documented as follows: 21.3°C with 1002.9 mm (autumn 2021), 19.4°C with 616.1 mm (spring 2022), and 21.7°C with 255.3 mm in autumn 2022 (**Figure 1**). The soil was classified as loamy sand; the soil chemical characteristics of 20 cm layer depth before the experiment were shown in **Table 1**. This presents a typical acidic soil with Mg deficiency prevalent in Southeast China.

**Table 1:** The soil chemical properties of 20 cm layer depth in 2021.

pH	Organic matter (g kg^−1^)	Alkali hydrolyzed N (mg kg^−1^)	Available P (mg kg^−1^)	Available K (mg kg^−1^)	Exchangeable Ca (mg kg^−1^)	Exchangeable Mg (mg kg^−1^)
4.28 ± 0.15	16.5 ± 1.3	163.7 ± 6.1	98.0 ± 3.4	218.0 ± 12.5	607.3 ± 20.4	47.7 ± 3.4

Values shown are the mean ± standard error (SE). P and Ca represent phosphorus and calcium, respectively.

### 2.2 Experiment design and field management

Field experiments were carried out using a randomized complete block design with three replicates. The experiment treatments were CK (foliar water spraying) and 2%, 4%, and 6% Mg (foliar spraying 2%, 4%, and 6% MgSO_4_·7H_2_O, respectively) in the 2021 autumn season. Based on the results of the 2021 experiment, subsequent experiments were conducted in the spring and autumn of 2022, and the experiment treatments were CK and 4% Mg (8.78 kg Mg ha^-1^). Mg fertilizer was sprayed on sweet corn leaf three times, at the V11 stage (11-leaf with visible leaf cushion) with 450 L ha^-1^, at the vegetative tasseling stage (VT), and at the silking stage (R1) of sweet corn with 900 L ha^-1^ once, and 0.1% Tween 20 was added in fertilizer solution. Each subplot was 48 m^2^ and remained unchanged for two years. Sweet corn cultivar Yongzhen7 (widely cultivated in Southeast China) was tested in our study, and the plant density was adopted with 60,000 plants ha^−1^, while the local conventional planting density was 45,000 plants ha^−1^. The cultivation schedule for cultivar Yongzhen7 was as follows: In the 2021 autumn season, seeds were sown on August 13, transplanted on August 25, and harvested on November 3. For the 2022 spring season, sowing occurred on March 17, transplantation on April 3, and harvesting on June 27. In the subsequent 2022 autumn season, sowing took place on August 12, transplantation on August 22, and the final harvest was conducted on November 1. 180 kg ha^−1^ N, 45 kg ha^−1^ P_2_O_5_, and 180 kg ha^−1^ K_2_O were applied in this study. Besides, fertilization methods, pest and weed control, and irrigation are carried out according to previous literature (Ye et al., 2023b).

### 2.3 Sampling and measurement

#### 2.3.1 Fresh ear yield and yield components

At the fresh eating stage (about 27 days after silking, R3 stage), the ears with cob and bract were hand-harvested from 30 consecutive sweet corn plants in each plot and weighed to calculate fresh ear yield. Subsequently, ear weight, ear bare tip length, 100-grain fresh weight, grains per ear, and grain abortion rate were measured following removing the bracts.

#### 2.3.2 Dry matter, nutrient accumulation, and soil plant analysis development value

Three key growth stages were selected for sampling: V12 (12-leaf with visible leaf cushion), R1 stage (silking stage), and R3 stage (fresh eating stage). The V12 stage marked the transition from vegetative to reproductive growth, initiating the differentiation of female flowers. The R1 stage was characterized by the emergence of silks from the husk leaves, indicating the onset of pollination. At the R3 stage, known as the fresh eating stage, the ears were harvested at their peak quality for consumption. At three critical stages, three sweet corn plants with consistent growth were sampled in each plot. The sampled plants were divided into stems (including leaf sheaths) and leaves at the V12 stage and divided the plant into three parts: stem (including leaf sheaths and tassel), leaves, and ear (including grains, cob, and bracts) at the R1 stage and R3 stage, and then dried samples at 70°C to a constant weight. The mineral element concentration of samples was determined. The total N and P concentrations were measured using a flow analyzer, total K was determined using a flame spectrophotometer, and Ca and Mg concentrations were measured using an inductively coupled plasma mass spectrometer (Li et al., 2021; Zhang et al., 2021b). Nutrient accumulation was measured by calculating N, P, K, Ca, and Mg accumulation in the different plant organs. Specifically, the nutrient accumulation in each organ (kg ha^-1^) was determined by multiplying the nutrient concentration in the organ by the organ’s dry weight. Total nutrient accumulation for each element (N, P, K, Ca, Mg) was then obtained by summing the nutrient accumulation across all plant organs. The ear leaf SPAD values of three representative plants in each plot were calculated using SPAD-502 Chlorophyll Meter at the V12, R1, and R3 stages.

#### 2.3.3 Grain filling rate and grain carbohydrate concentration

In each plot, 40 representative and consistent sweet corn female ears were labelled at the silking stage. At least five ears were collected at 7, 12, 17, 22, and 27 days after silking. The grains from the middle ear were sampled and divided into two parts. One part of the grain was weighed for fresh weight and then dried to determine the grain’s dry weight. The other fresh grains were collected and grinded with liquid N to determine the concentrations of grain sucrose, fructose, and starch (Li and Li, 2013). Glucose concentrate in sweet corn grain was determined using the detection kit (UPLC-MS-4105) provided by Shanghai Liquid Quality Detection Technology Co., Ltd. The grain filling rate was the increase in grain dry weight per unit time.

### 2.4 Statistical analysis

Drawings were carried out using Origin 2023 software. Multiple comparisons were performed using Duncan’s test at the *P* < 0.05 level in 2021, and the differences between treatments in 2022 were analyzed using Student’s t-tests with the SPSS software (version 22.0, SPSS, Chicago, IL, USA). The Pearson correlation analysis method was used to perform correlation analysis.

## 3 Results

### 3.1 Yield and yield components

**Figure 2:**
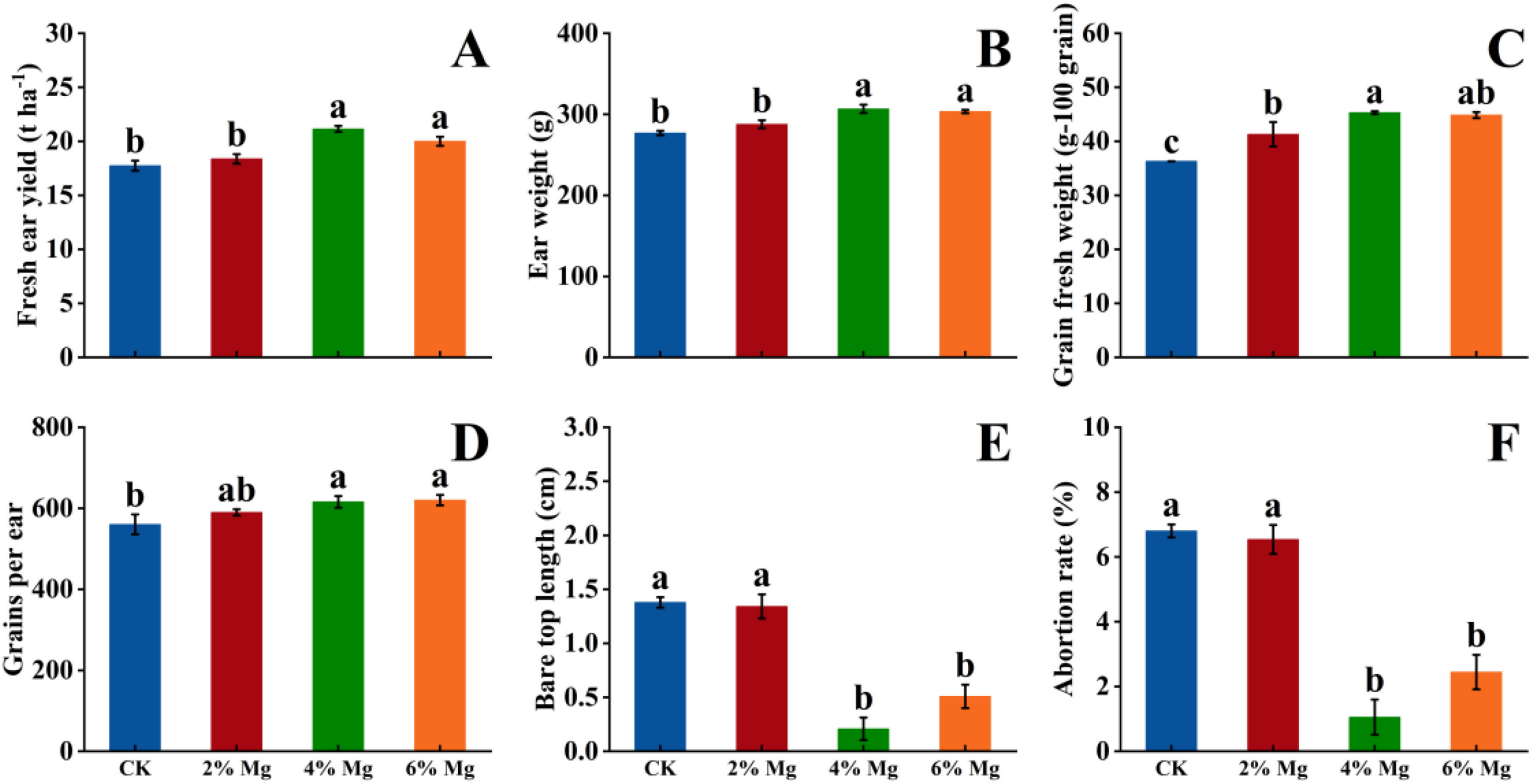
Effects of foliar application of magnesium fertilizer on fresh ear yield **(A)**, ear weight **(B)**, grain fresh weight **(C)**, grains per ear **(D)**, bare tip length **(E)**, and abortion rate **(F)** of sweet corn in 2021. Data are presented as treatments mean ± SE, data followed by same letter are not significantly different at *P* < 0.05.

**Figure 3:**
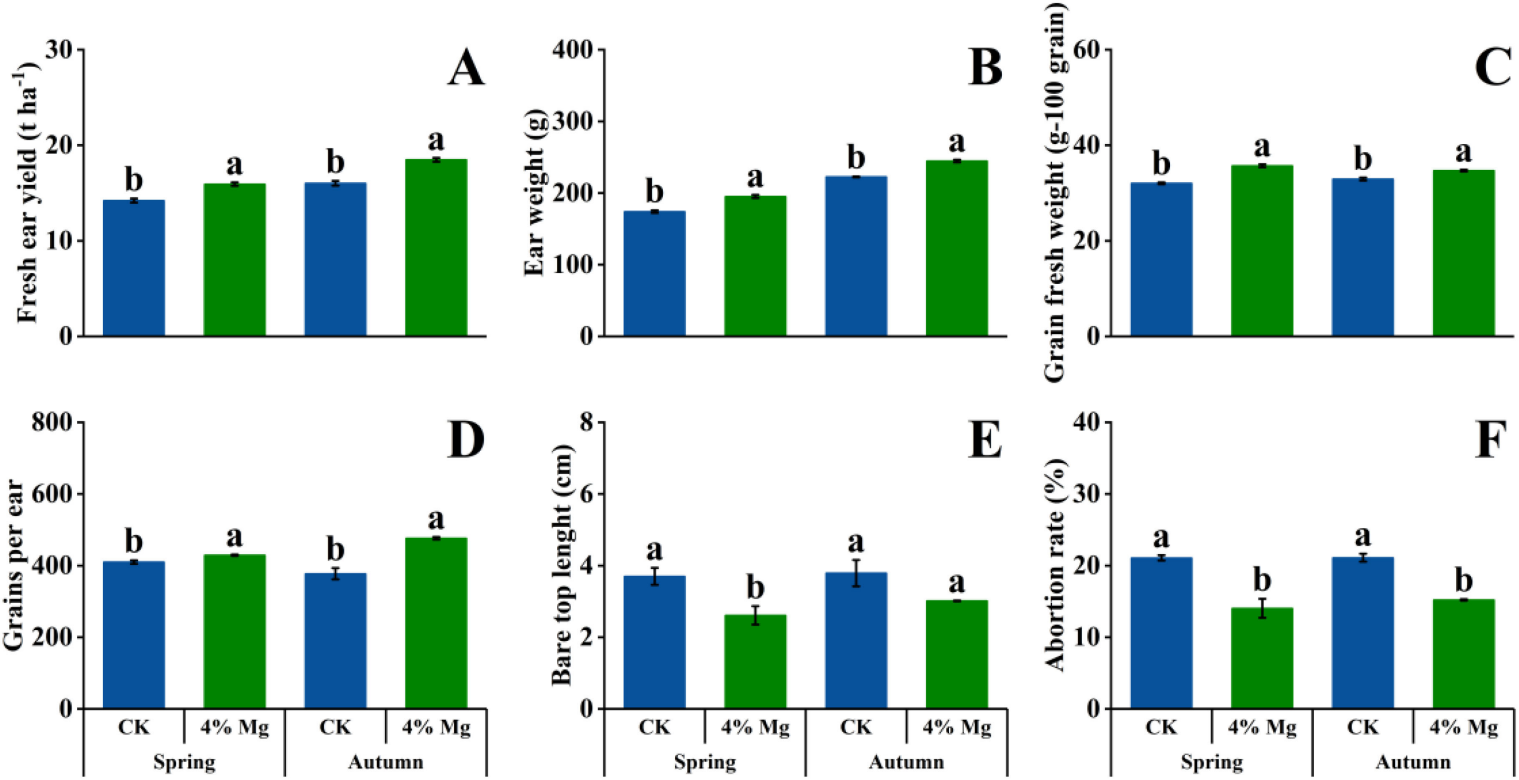
Effects of foliar application of magnesium fertilizer on fresh ear yield **(A)**, ear weight **(B)**, grain fresh weight **(C)**, grains per ear **(D)**, bare tip length **(E)**, and abortion rate **(F)** of sweet corn in spring and autumn seasons growing season of 2022. Data are presented as treatments mean ± SE, data followed by the same letter are not significantly different between CK and 4% Mg treatments at *P* < 0.05.

Compared to CK treatment, foliar spraying of 4% Mg and 6% Mg significantly increased fresh ear yield, ear weight, and grains per ear but significantly decreased bare tip length and abortion rate in the autumn growing season of 2021. Foliar spraying Mg fertilization significantly increased grains fresh weight. However, no marked differences existed between 4% Mg and 6% Mg treatment for fresh ear yield and yield components in the autumn growing season 2021 (**Figure 2**). In addition, 4% Mg treatment significantly increased sweet corn fresh ear yield, ear weight, grains fresh weight, and grains per ear while significantly decreased the abortion rate compared with CK treatment in the spring and autumn growing seasons of 2022 (**Figure 3**). These results indicated that the optimum foliar Mg concentration was 4%.

### 3.2 Dry matter and nutrient accumulation

**Figure 4:**
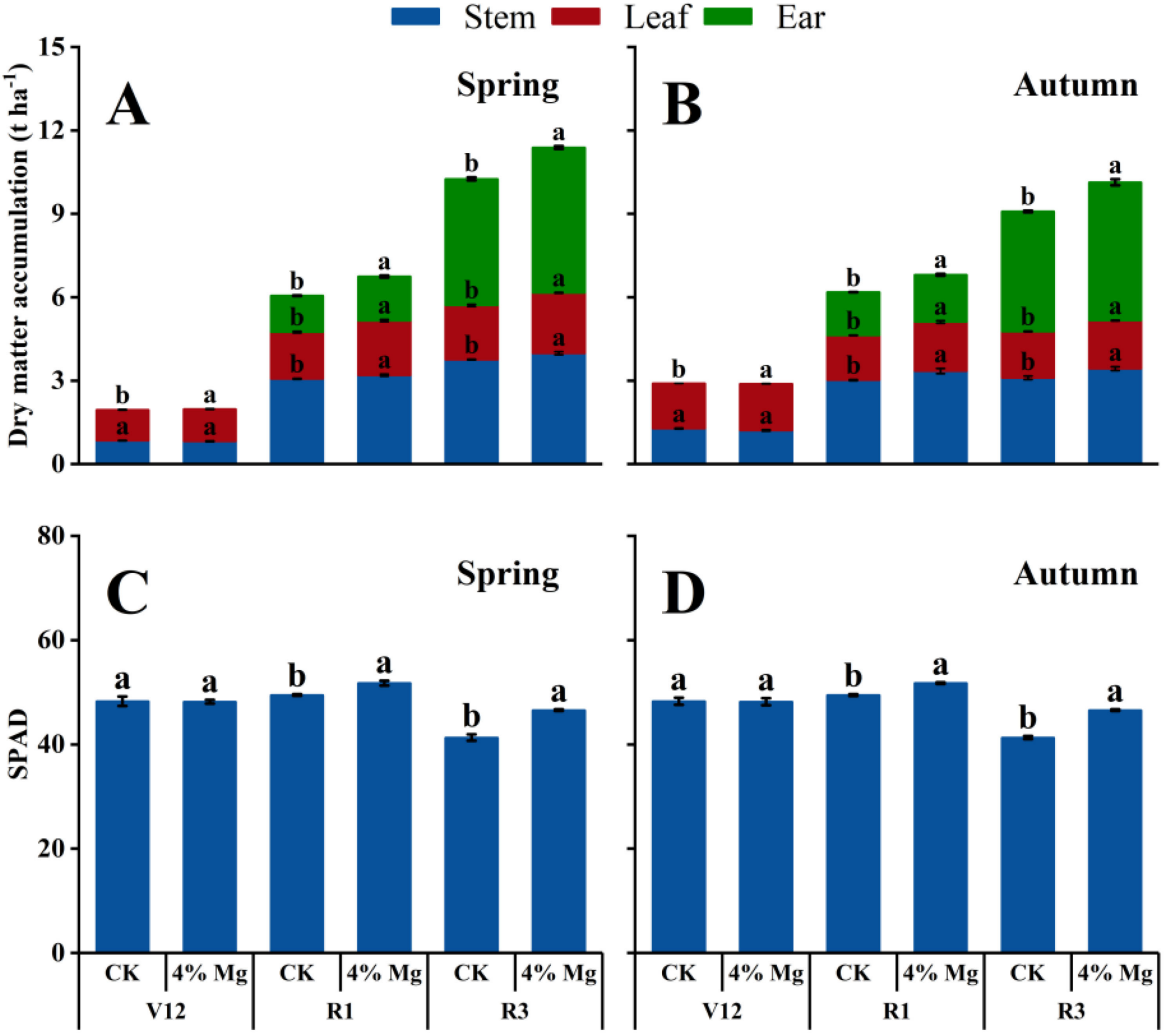
Effect of foliar application of magnesium fertilizer on the dry matter **(A, B)** and SPAD **(C, D)** of sweet corn in the spring and autumn growing season of 2022. Data are presented as treatments mean ± SE; data followed by the same letter are not significantly different between CK and 4% Mg treatments at *P* < 0.05.

Compared to CK treatment, foliar spraying 4% Mg markedly increased stem, leaf, ear, and total dry matter of sweet corn at R1 and R3 growth stages in both seasons of 2022. The total dry matter in the spring and autumn seasons under 4% Mg treatment was 11.0% and 11.5% higher than those under CK treatment, respectively. 4% Mg treatment significantly increased SPAD at R1 and R3 growth stages in both seasons, increased by an average of 6.9% and 9.3% compared to CK treatment, respectively. However, no prominent increase exited in SPAD at the V12 stage between CK and 4% Mg treatment (**Figure 4**).

Foliar spraying of 4% Mg significantly increased N concentration and N accumulation in the stem, leaf, and ear at the R3 stage. Foliar spraying of 4% Mg significantly increased P, K, and Ca accumulation but did not significantly affect P, K, and Ca concentrations in most organs (**Table 2**).

**Table 2:** Effect of foliar magnesium application on N, P, K, Ca concentration and accumulation of sweet corn in spring and autumn of 2022.

Nutrient	Season	Treatment	Concentration (g kg^-1^)			Accumulation (kg ha^-1^)			Total
			Stem	Leaf	Ear	Stem	Leaf	Ear	
N	Spring	CK	6.9 ± 0.03 b	16.77 ± 0.16 b	13.44 ± 0.16 b	25.92 ± 0.2 b	32.04 ± 1.01 b	61.15 ± 0.81 b	119.11 ± 1.3 b
		4% Mg	7.69 ± 0.17 a	17.73 ± 0.26 a	13.75 ± 0.16 a	30.68 ± 1.08 a	37.87 ± 0.72 a	71.8 ± 0.25 a	140.35 ± 1.57 a
	Autumn	CK	6.91 ± 0.08 b	15.55 ± 0.24 b	12.68 ± 0.48 b	21.44 ± 0.59 b	25.96 ± 0.64 b	54.74 ± 2.32 b	102.14 ± 1.83 b
		4% Mg	7.65 ± 0.2 a	17.16 ± 0.4 a	13.47 ± 0.46 a	26.23 ± 0.62 a	29.78 ± 0.76 a	66.96 ± 3.08 a	122.97 ± 4.04 a
P	Spring	CK	1.36 ± 0.02 a	2.61 ± 0.02 a	3.42 ± 0.12 a	5.13 ± 0.09 a	5.08 ± 0.07 a	15.54 ± 0.56 b	25.75 ± 0.63 b
		4% Mg	1.38 ± 0.02 a	2.61 ± 0.04 a	3.52 ± 0.03 a	5.5 ± 0.13 a	5.68 ± 0.11 a	18.4 ± 0.19 a	29.58 ± 0.32 a
	Autumn	CK	1.64 ± 0.02 a	2.54 ± 0.03 a	3.54 ± 0.12 a	5.09 ± 0.17 b	4.24 ± 0.02 a	15.25 ± 0.44 a	24.59 ± 0.27 a
		4% Mg	1.67 ± 0.02 a	2.55 ± 0.02 a	3.63 ± 0.29 a	5.71 ± 0.08 a	4.43 ± 0.06 a	18.12 ± 1.88 a	28.26 ± 1.99 a
K	Spring	CK	22.61 ± 0.4 a	17.08 ± 0.4 a	7.74 ± 0.11 a	84.97 ± 1.2 b	33.32 ± 1.39 b	35.2 ± 0.41 b	153.49 ± 2.52 b
		4% Mg	22.71 ± 0.23 a	17.65 ± 0.23 a	7.63 ± 0.2 a	90.56 ± 1.62 a	38.44 ± 0.8 a	39.84 ± 0.69 a	168.84 ± 1.69 a
	Autumn	CK	24.31 ± 0.05 b	17.95 ± 0.14 a	8.14 ± 0.16 a	75.42 ± 1.65 b	29.95 ± 0.15 b	35.12 ± 0.91 b	140.49 ± 2.28 b
		4% Mg	24.93 ± 0.19 a	18.59 ± 0.21 a	8.41 ± 0.02 a	85.47 ± 1.08 a	32.27 ± 0.57 a	41.78 ± 1.01 a	159.52 ± 1.73 a
Ca	Spring	CK	7.17 ± 0.02 a	12.51 ± 0.44 a	0.95 ± 0.07 a	26.94 ± 0.17 b	24.42 ± 1.28 b	4.34 ± 0.29 a	55.7 ± 1.02 b
		4% Mg	7.28 ± 0.05 a	13.36 ± 0.26 a	0.96 ± 0.02 a	29.01 ± 0.28 a	29.08 ± 0.43 a	5.01 ± 0.16 a	63.1 ± 0.25 a
	Autumn	CK	9.3 ± 0.03 a	12.15 ± 0.44 a	1.03 ± 0.01 b	28.83 ± 0.47 a	20.27 ± 0.7 a	4.45 ± 0.03 b	53.55 ± 0.98 b
		4% Mg	9.51 ± 0.31 a	12.53 ± 0.25 a	1.06 ± 0 a	32.65 ± 1.61 a	21.75 ± 0.52 a	5.25 ± 0.1 a	59.66 ± 1.72 a
Variation of source									
N	Mg		***	**	ns	***	***	***	***
	Season		ns	*	ns	***	***	*	***
	Mg × Season		ns	ns	ns	ns	ns	ns	ns
P	Mg		ns	ns	ns	**	**	*	**
	Season		***	ns	ns	ns	***	ns	ns
	Mg × Season		ns	ns	ns	ns	*	ns	ns
K	Mg		ns	ns	ns	**	**	***	***
	Season		***	**	**	**	**	ns	**
	Mg × Season		ns	ns	ns	ns	ns	ns	ns
Ca	Mg		ns	ns	ns	**	**	**	***
	Season		***	ns	*	**	***	ns	*
	Mg × Season		ns	ns	ns	ns	ns	ns	ns

Values are presented as mean ± SE; values followed by the same letter are not significantly different between CK and 4% Mg treatments at *P* < 0.05. *, ** and *** indicate signiﬁcances at *P* < 0.05, *P* < 0.01 and *P* < 0.001, respectively. ns indicate no significance (*P* > 0.05).

### 3.3 Mg uptake and remobilization

**Figure 5:**
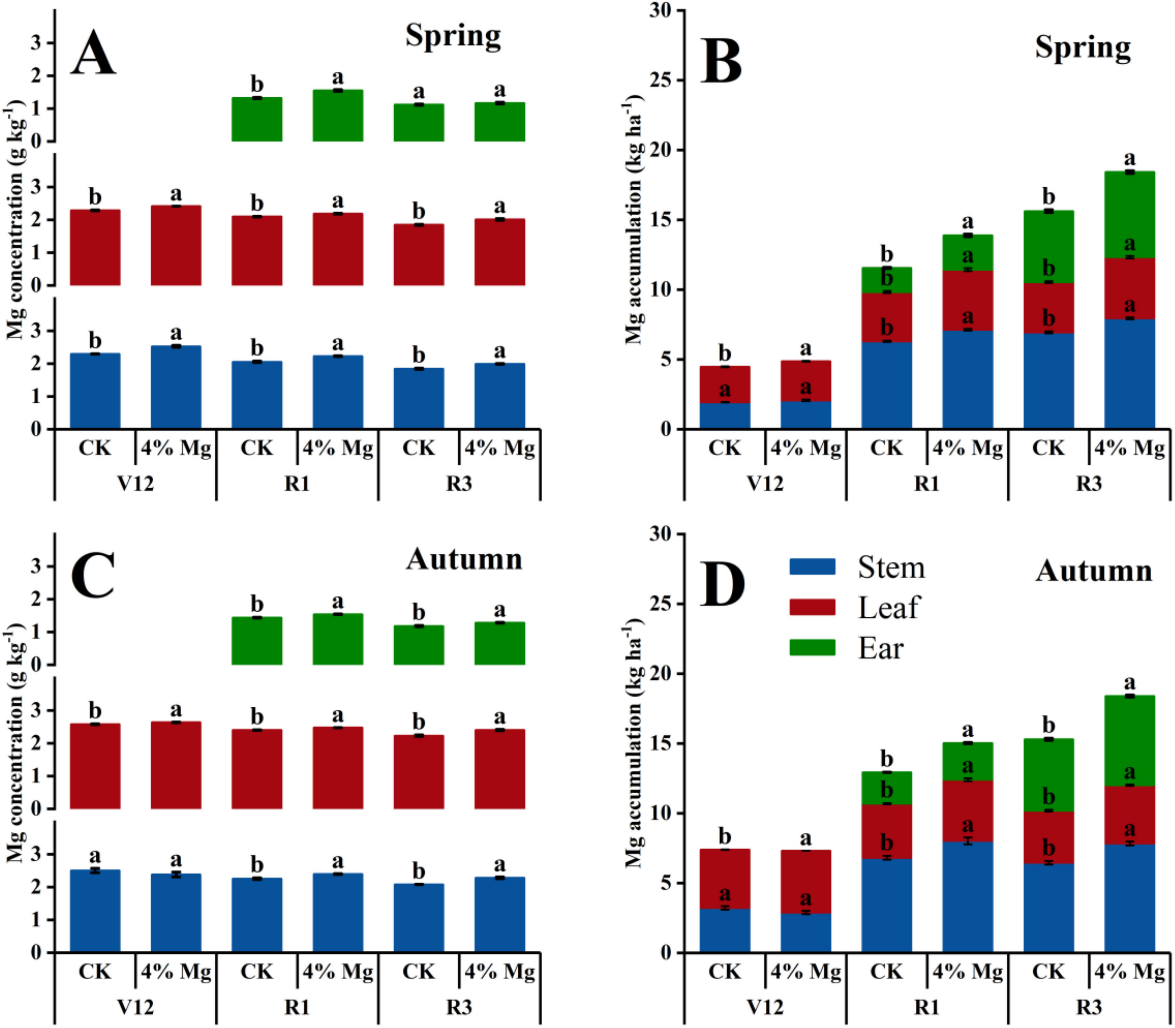
Effect of foliar application of magnesium fertilizer on the Mg concentration **(A, C)** and Mg accumulation **(B, D)** of sweet corn in the spring and autumn growing season of 2022. Data are presented as treatments mean ± SE; data followed by the same letter are not significantly different between CK and 4% Mg treatments at *P* < 0.05.

As the growth stage advanced, sweet corn Mg concentration in different organs gradually decreased, but Mg accumulation increased (**Figure 5**). Foliar spraying of 4% Mg significantly increased Mg concentration in stem, leaf, and ear of sweet corn at V12, R1, and R3 stages in both seasons for 2022, except for ear Mg concentration at the R3 stage and stem Mg concentration at V12 stage (**Figure 5**). Foliar spraying of 4% Mg significantly increased Mg accumulation in stem, leaf, and ear at V12, R1, and R3 stages in both seasons, except for stem Mg accumulation at the V12 stage. The total Mg accumulations at the R3 stage in spring and autumn seasons under 4% Mg were 17.9% and 20.2% higher than those under CK treatment. In addition, the Mg uptake rate from the V12 stage to the silking stage was the biggest during different growing stages in sweet corn (**Figure 5**).

Foliar 4% Mg significantly increased sweet corn Mg accumulation at pre-silking in both seasons and significantly increased Mg accumulation at post-silking in the autumn season. Moreover, the Mg accumulation of sweet corn was an average of 16.9 kg ha^-1^ and 21.1% of total Mg absorbed after silking. However, the Mg remobilization amount from the stems and leaves was limited (< 5% of total Mg accumulation), and the contribution rate of Mg remobilization from stem and leaf to ear was < 10% (**Table 3**).

**Table 3:** Effect of foliar application of magnesium fertilizer on Mg accumulation, Mg remobilization amount, and contribution rate of Mg remobilization to ear of sweet corn in spring and autumn of 2022.

Season	Treatment	Mg accumulation (kg ha^-1^)		Mg remobilization amount (kg ha^-1^)		Contribution rate of Mg remobilization to ear (%)	
		Pre-silking	Post-Silking	Stem	Leaf	Stem	Leaf
Spring	CK	11.6 ± 0.07 b	4.1 ± 0.12 a	-0.64 ± 0.04 a	-0.07 ± 0.02 a	-12.68 ± 1.00 a	-1.38 ± 0.31 a
	4% Mg	13.9 ± 0.12 a	4.5 ± 0.26 a	-0.82 ± 0.02 b	-0.07 ± 0.04 a	-13.49 ± 0.13 a	-1.20 ± 0.70 a
Autumn	CK	12.9 ± 0.11 b	2.4 ± 0.09 b	0.35 ± 0.01 a	0.15 ± 0.04 a	6.84 ± 0.06 a	2.98 ± 0.80 a
	4% Mg	15.0 ± 0.26 a	3.4 ± 0.18 a	0.19 ± 0.21 a	0.20 ± 0.06 a	2.86 ± 3.26 a	3.21 ± 1.00 a
Variation of source							
Mg		***	**	ns	ns	ns	ns
Season		***	***	***	***	***	***
Mg × Season		ns	ns	ns	ns	ns	ns

Values are presented as mean ± SE; values followed by the same letter are not significantly different between CK and 4% Mg treatments at *P* < 0.05. ** and *** indicate signiﬁcances at *P* < 0.01 and *P* < 0.001, respectively. ns indicate no significance (*P* > 0.05).

### 3.4 Grain filling rate and grain carbohydrate concentration

**Figure 6:**
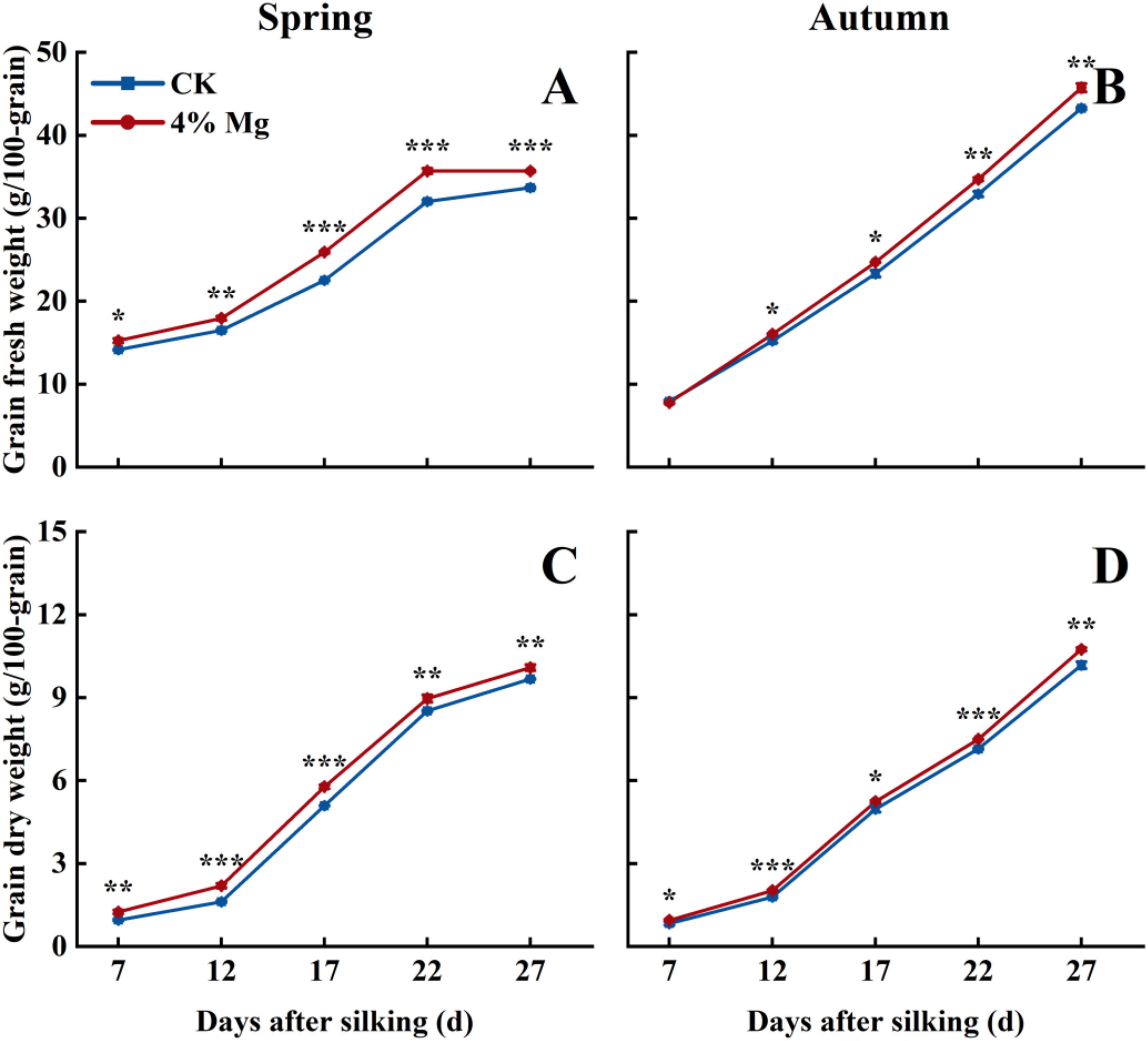
Effect of foliar application of magnesium fertilizer on grain fresh weight **(A, B)** and kernel dry weight **(C, D)** of sweet corn in spring and autumn growing season of 2022. The error bar indicates SE. *, *P* < 0.05; **, *P* < 0 01; ***, *P* < 0.001, according to Student’s t-test.

**Figure 7:**
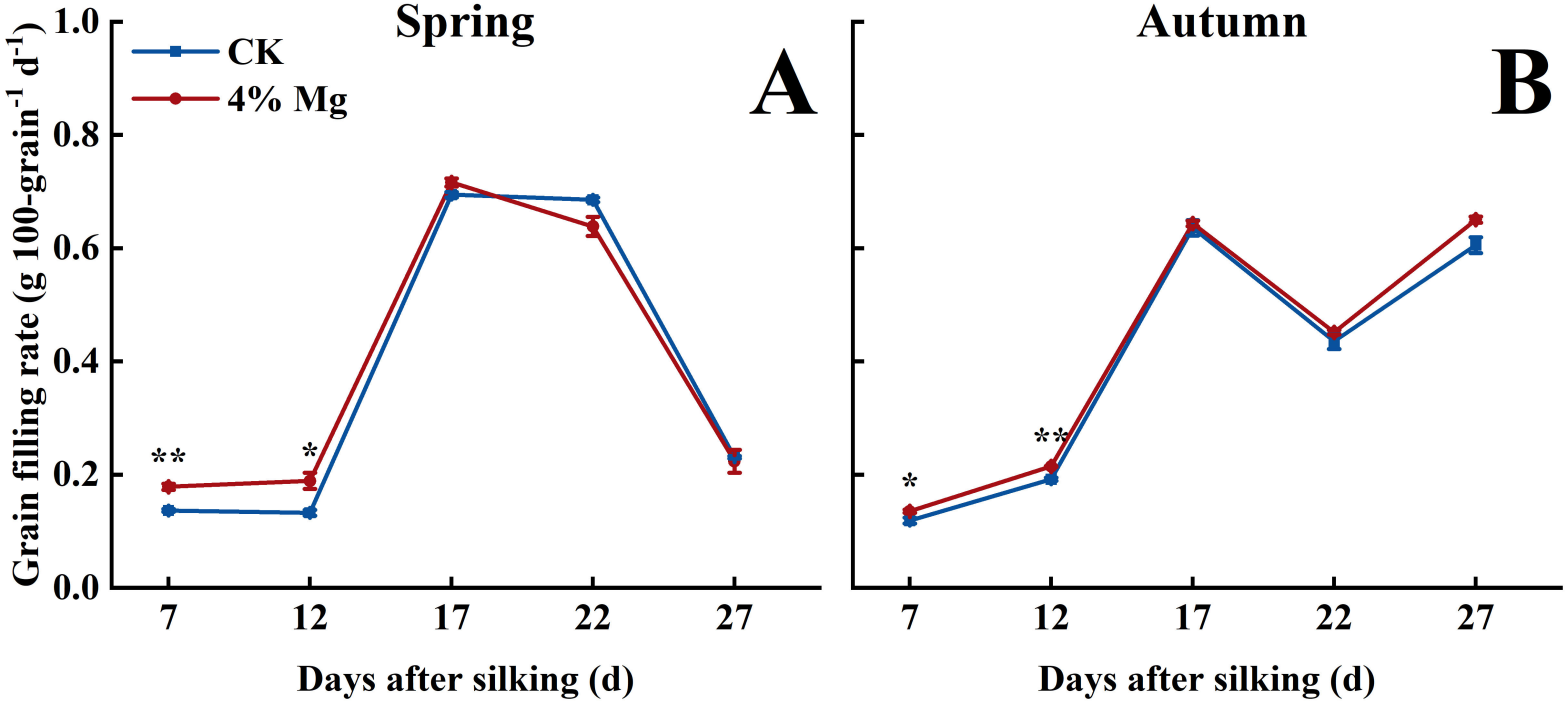
Effect of foliar application of magnesium fertilizer on grain filling rate of sweet corn in spring and autumn growing season of 2022. The error bar indicates SE. *, *P* < 0.05; **, *P* < 0 01, according to Student’s t-test.

Sweet corn grain’s fresh weight and dry weight gradually increased with the advancement of grain filling. Foliar spraying of 4% Mg significantly increased grain fresh weight and dry weight at 12, 17, 22, and 27 days after silking in both growing seasons (**Figure 6**). The grain filling rate reached its maximum value at 17 days after silking, and 4% Mg treatment significantly increased the grain filling rate at 7 and 12 days in the spring and autumn growing seasons. However, it did not markedly affect the grain filling rate at 17–27 days (**Figure 7**).

**Figure 8:**
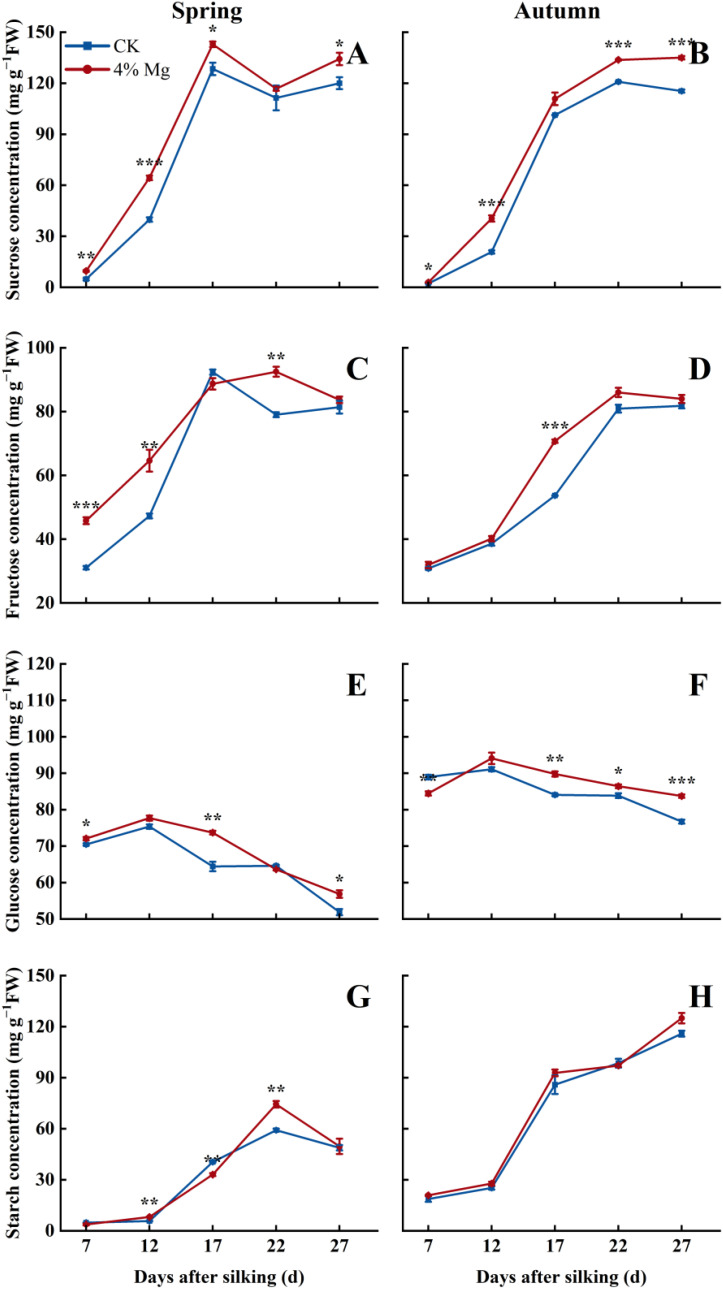
Effect of foliar application of magnesium fertilizer on sucrose concentration **(A, B)**, fructose concentration **(C, D)**, glucose concentration **(E, F)**, and starch concentration **(G, H)** of sweet corn in spring and autumn growing season of 2022. The error bar indicates SE. *, *P* < 0.05; **, *P* < 0 01; ***, *P* < 0.001, according to Student’s t-test.

Sweet corn grain sucrose, fructose, and starch concentrations were increased with the advance of grain filling. However, grain glucose concentration was first increased and then decreased (**Figure 8**). 4% of Mg treatment significantly increased grain sucrose, fructose, and glucose concentrations during the grain filling period in both seasons; the 4% of Mg treatment average increased by 14.4%, 2.7%, and 9.3% at 27 days after silking compared to CK treatment. 4% Mg treatment dramatically increased grain starch concentration in spring but did not significantly enhance grain starch concentration in autumn (**Figure 8**).

### 3.5 The relationship between fresh ear yield and other traits

**Figure 9:**
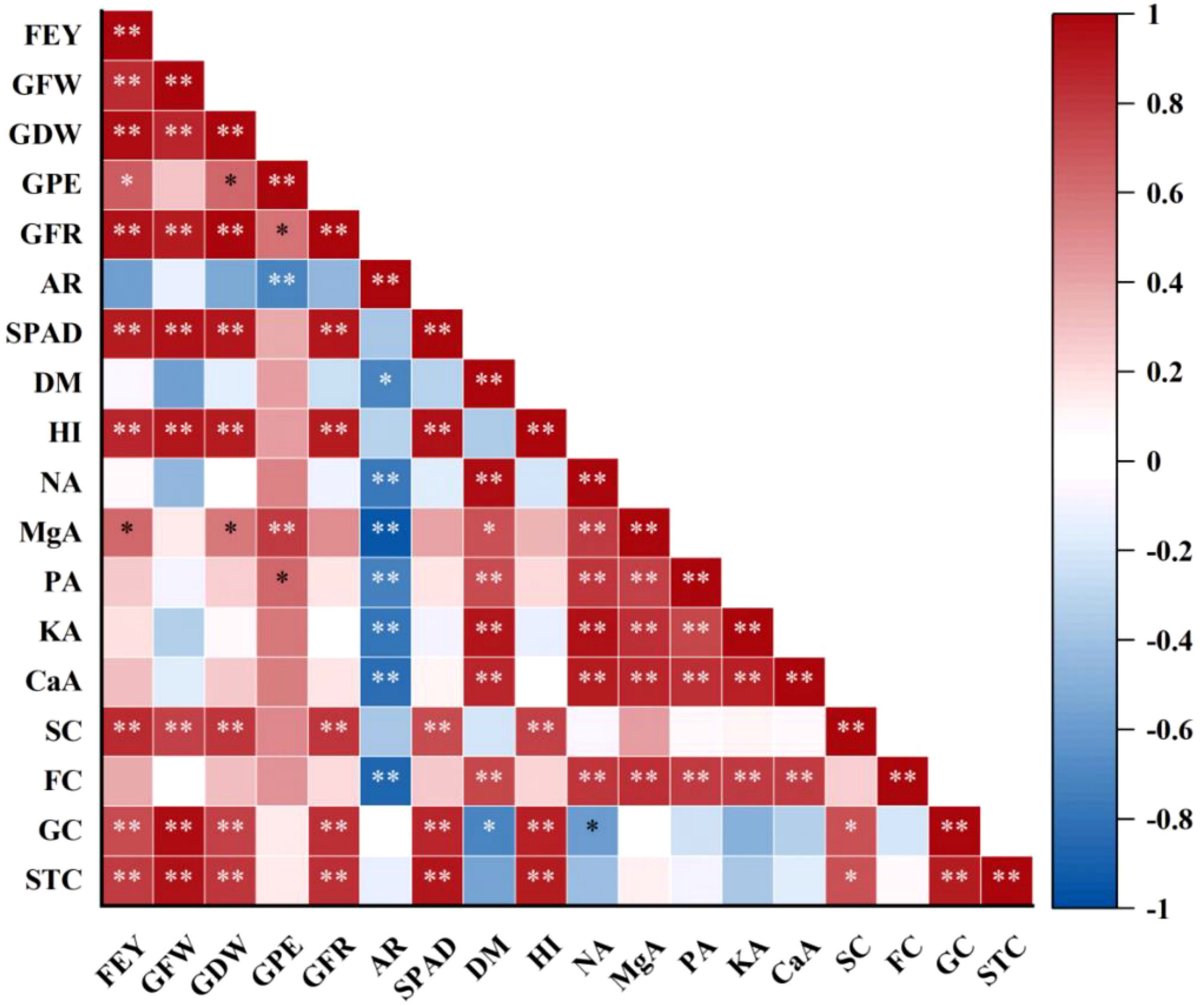
Correlation among fresh ear yield (FEY), grain fresh weight (GFW), grain dry weight (GDW), grains per ear (GPE), grain filling rate (GFR), abortion rate (AR), SPAD, dry matter accumulation (DM), harvest index (HI), N accumulation (NA), Mg accumulation (MgA), P accumulation (PA), K accumulation (KA), Ca accumulation (CaA), sucrose concentration (SC), fructose concentration (FC), glucose concentration (GC) and starch concentration (STC). * and ** indicate significant correlation at *P* < 0.05, *P* < 0.01, respectively.

Sweet corn fresh ear yield was significantly positively correlated with grain dry weight (r = 0.96), grains per ear (r = 0.67), grain filling rate (r = 0.95), SPAD value (r = 0.91), harvest index (r = 0.87), Mg accumulation (r = 0.64), sucrose concentration (r = 0.85), glucose concentration (r = 0.73) and starch concentration (r = 0.78). Moreover, Mg accumulation was significantly positively related to grain dry weight (r = 0.58), grains per ear (r = 0.80), N accumulation (r = 0.80), P accumulation (r = 0.77), K accumulation (r = 0.84), Ca accumulation (r = 0.88) and fructose concentration (r = 0.83) (**Figure 9**).

## 4 Discussion

Abiotic stress, such as dense planting, has significantly contributed to yield loss in corn, as it reduces the number of grains per ear and grain weight (Ye et al., 2023b). This stress leads to nutritional competition, grain abortion, and lodging, all of which impede yield potential (Sher et al., 2017; Shah et al., 2021). Strategic nutrient management, particularly Mg supplementation, becomes critical under high planting conditions, given Mg’s central role in enzyme activation, energy transfer, and nutrient metabolism (Tian et al., 2021). The urgency for Mg optimization is heightened in southern China’s acidic soils, where 55% of arable lands exhibit Mg deficiency-a key constraint for high-yield cropping systems (Ishfaq et al., 2022). In field corn production in Brazil, foliar application of only 0.5–1 kg ha^-1^ Mg has been shown to significantly increase both Mg concentration and grain yield (Altarugio et al., 2017; Rodrigues et al., 2021). Nevertheless, foliar applying 3.5 kg ha^-1^ Mg significantly increases organ Mg concentration and yield in wheat (Kibria et al., 2021). These inconsistent reports indicate that the effects of foliar spraying Mg depend on crop type and environmental condition, so it is necessary and important to note the effect of reasonable foliar Mg on sweet corn in Southeast China. In this study, foliar spraying 8.78 kg ha^-1^ Mg significantly increased sweet corn fresh ear yield, grains per ear, and grain weight. Szulc et al. (2011) also found that foliar application of 6 kg ha^-1^ Mg significantly field corn yield and grain weight. This can be attributed to several factors: first, Mg enhances the photosynthetic efficiency by optimizing photosystem activity and improving carbon fixation rates. Additionally, it plays a critical role in activating enzymes involved in starch synthesis, thereby enhancing carbohydrate metabolism and contributing to yield formation (Cakmak and Kirkby, 2008; Ceylan et al., 2016; Ba et al., 2020). Second, Mg improves abiotic stress tolerance to by boosting antioxidant enzyme activity and delaying leaf senescence, particularly under high-density planting conditions (Rodrigues et al., 2021). It also improves cell membrane thermostability and pollen viability, both of which are vital for maintaining cellular integrity and reproductive success, leading to increased grain weight and yield (Raza et al., 2023). Third, Mg promotes root growth and upregulates the nitrate transporter genes expression, thereby increasing N uptake, assimilation, and use efficiency, which further contributes to increased yield (Peng et al., 2020; Lyu et al., 2023). In this study, sweet corn fresh ear yield was significantly positively correlated with harvest index but not biomass, indicating that the ability to allocate biomass to the ear plays a more significant role in yield formation than total biomass accumulation. This finding is consistent with previous research of Djaman et al. (2013). Notably, grains per ear, grain weight, and abortion rate exhibited season-specific responses, likely influenced by temperature and precipitation during grain-filling. Further multi-year trials under varying environmental conditions are necessary to confirm these observations and gain a deeper understanding of the underlying mechanisms.

Nutrient uptake exhibits a significant positive correlation with crop yield, predominantly regulated by fertilization strategies (Setiyono et al., 2010). In this study, foliar spraying Mg application increased N, P, K, Ca, and Mg accumulation of sweet corn, aligning with prior reports observations of Mg-induced nutrient enrichment in field corn (Zhang et al., 2021a; El-Dissoky et al., 2017). It may be due to the contribution of Mg to chlorophyll formation and photosynthesis, producing more dry matter, thereby driving more N uptake (Peng et al., 2010; Ahmed et al., 2020). Additionally, Mg can increase root length and dry weight, acquiring more nutrients (Kong et al., 2020). In particular, foliar Mg fertilizer significantly increased Mg and N concentration in the organs of sweet corn but did not affect P, K, and Ca concentrations in this study. Similar results are demonstrated under field corn in the Mediterranean region (Ortas, 2018). However, Ahmed et al. (2020) highlighted that Mg supplementation significantly decreased K concentration, although improving Mg and N concentrations of field corn in calcareous soils. These discrepancies suggest that foliar Mg circumvents root-level Mg-K/Ca antagonism and soil Mg-P precipitation (Grzebisz, 2013; Xie et al., 2021), highlighting its precision in dense planting systems. Moreover, the synergistic Mg-N relationship is mechanistically driven by two factors: (1) Mg activation of Mg²^+^-ATPase and glutamine synthetase, which catalyzes NH_4_^+^ assimilation (Jezek et al., 2015a); and (2) Mg-induced upregulation of N transporter genes, enhancing N uptake and utilization efficiency (Peng et al., 2020). Unlike soil application, which supports a long-term nutrient supply, foliar fertilization can only provide a limited quantity of nutrients and is sensitive to environmental factors (Ssemugenze et al., 2025). However, it offers a rapid method for nutrient delivery, especially during critical growth stages, by bypassing soil limitations such as low nutrient availability and ion antagonism (Niu et al., 2021). Therefore, combining foliar and soil application of Mg fertilization may ensure immediate and long-term availability, ultimately improving corn growth and yield.

Understanding Mg’s dynamic accumulation and distribution characteristics is an essential foundation for the scientific application of Mg fertilizer (Chen et al., 2016). Mg accumulation of sweet corn was an average of 16.9 kg ha^-1^ and 21.1% of total Mg absorbed after silking in the current study. Similar results were reported in field corn: a total accumulation of approximately 33 kg ha^-1^ Mg, with 23%-30% accumulating after silking (Ciampitti and Vyn, 2013). However, Zhang et al. (2020) documented that the total Mg accumulation was over 120 kg ha^-1,^and 44.2% of Mg accumulation was obtained at the reproductive stage of field corn in Northeast China. This may be due to differences in variety, cultivation practices, and environmental conditions. In addition, in this study, sweet corn Mg remobilization from vegetative organs was < 5% of total Mg accumulation, and contribution of Mg remobilization to ear was < 10%. This is inconsistent with the results showing that Mg’s remobilization efficiency from stalk was 23%, while the leaf Mg remobilization efficiency was -36% in field corn (Chen et al., 2016). However, Mg remobilization from the stalk happens 30 days after silking in field corn (Chen et al., 2016), and sweet corn ears are harvested 27 days after silking. This indicates that the Mg requirement of the ear in sweet corn primarily relies on absorption after silking, rather than on translocation from vegetative organs. The limited Mg transfer to the ear may be attributed to restricted sink capacity, which hinders Mg mobilization from the stems and leaves to the ear (Szczepaniak et al., 2016). Furthermore, genetic differences among cultivars significantly influence Mg transport efficiency, with low Mg transport efficiency in sweet corn limiting the extent of Mg translocation (Kovacevic et al., 2004). However, the approach used to study Mg remobilization in our research has certain limitations. Future work should employ isotope tracing techniques to accurately quantify Mg remobilization and its contribution to the ear. Furthermore, the Mg uptake rate from V12 to the silking stage was the biggest during different growing stages in sweet corn, which agreed with the previous study’s finding (Zhang et al., 2020). Therefore, V12 - silking are the critical stages for Mg fertilizer application. These results provide a clear understanding of Mg uptake and remobilization dynamics in sweet corn, particularly during the post-silking period. These findings suggest that foliar application of Mg (total 8.78 kg Mg ha^-1^) during the mid-to-late growth stages is an effective strategy to meet Mg demand and optimize grain filling. This provides a practical and scientific foundation to improve Mg fertilization efficiency in sweet corn cultivation.

Grain filling is a process of grain accumulation and storing photosynthetic products, a critical stage affecting yield (Li et al., 2020). Grain filling rate has a significant positive correlation with yield and grain dry weight (Ye et al., 2023b), similar results were shown in this study. Dense planting usually reduces grain filling rate (Ye et al., 2023b); however, foliar spraying Mg significantly sweet corn leaf SPAD and grain filling rate in the early stage of grain filling under close planting in this study. One explanation is that Mg application can delay leaf senescence and produce more photosynthetic products during grain filling, subsequently transferring more photosynthetic products to the grain, increasing the grain filling rate (Jezek et al., 2015b; Ning et al., 2018; Hauer-Jákli and Tränkner, 2019). While our findings suggest that improving the grain filling rate under high-density planting is critical for yield enhancement, the underlying physiological and molecular mechanisms remain unclear and require further investigation.

Grain carbohydrates, including sucrose, fructose, glucose, and starch, are vital components that determine grain weight and yield. Hence, understanding the dynamic changes of carbohydrate components is an important way to improve crop yield and quality (Ye et al., 2023b; Paponov et al., 2005). Sucrose, fructose, and starch concentration gradually increased as the grain-filling progresses, which is agreed with the previous literature (Paponov et al., 2005). However, glucose concentration was first increased and then decreased in this study. It may be due to glucose transfer to another form of carbohydrates during sweet corn grain filling. In the current investigation, Mg significantly increased grain carbohydrates (sucrose, fructose, and starch) concentration. Ba et al. (2020) also stated that foliar application of Mg fertilizer enhanced grain carbohydrate concentration in wheat. This may be attributed to Mg’s role in facilitating long-distance carbohydrate transport (Jiao et al., 2023), enhancing the activity of key enzymes and gene expression involved in carbohydrate metabolism (Ba et al., 2020; He et al., 2024a, 2024b). Mg also promotes the synthesis of abscisic acid (ABA) and the activity of catalase (CAT), which are known to regulate grain carbohydrate accumulation (He et al., 2024a). These mechanisms highlight how Mg influences carbohydrate transport and metabolism, ultimately contributing to improved carbohydrate availability for grain development. Carbohydrate metabolism plays a critical role in determining yield and grain quality in sweet corn. However, the mechanisms that foliar Mg regulates carbohydrate dynamics, particularly its effects on key enzymes and transcriptional networks involved in starch and sucrose biosynthesis, remain unclear. Future studies should focus on elucidating how Mg regulates carbohydrate metabolism both at the biochemical and transcriptional levels, with a particular emphasis on rate-limiting enzymes and associated gene pathways involved in carbohydrate accumulation (Tahir et al., 2020; Shen et al., 2022). Although conventional planting density treatments (45,000 plants ha^−^¹) were not included in the current study, future investigations should implement multi-density field trials to mechanistically assess how planting density regulates the Mg uptake and translocation process.

## 5 Conclusion

Foliar spraying of 4% Mg significantly increased sweet corn fresh ear yield, grains per ear, and grain weight under 60,000 plants ha^−1^ over three seasons in 2021 and 2022. It also markedly increased dry matter, N, K, Ca, and Mg accumulation, as well as the concentrations of sucrose, fructose, and glucose in the grains during the spring and autumn growing seasons of 2022. Therefore, foliar spraying of 4% MgSO_4_·7H_2_O three times (total 8.78 kg Mg ha^-1^) can increase yield, nutrient uptake, and grain sugar concentrations, making it a recommended method for Mg application in sweet corn. This finding establishes a scientifically optimized Mg fertilization protocol for intensive sweet corn cultivation, demonstrating that targeted foliar supplementation can mitigate nutrient competition in high-density cropping.

## Data Availability

The original contributions presented in the study are included in the article/Supplementary Material. Further inquiries can be directed to the corresponding author.
